# Comparison of C2 dome-like laminectomy with C2 partial laminectomy for upper cervical ossification of the posterior longitudinal ligament

**DOI:** 10.3389/fsurg.2022.1087157

**Published:** 2023-01-09

**Authors:** Dazhuang Miao, Xianda Gao, Zihao Zhen, Dalong Yang, Hui Wang, Wenyuan Ding

**Affiliations:** Department of Spine Surgery, The Third Hospital of Hebei Medical University, Shijiazhuang, China

**Keywords:** OPLL, cervical spine, c2 partial laminectomy, c2 dome-like laminectomy, axial symptoms

## Abstract

**Objective:**

To compare surgical outcomes of C2 dome-like laminectomy with C2 partial laminectomy in patients with ossification of the posterior longitudinal ligament (OPLL) up to the C2 level and above.

**Methods:**

32 patients underwent surgical treatment for OPLL up to C2 and were divided into: C2 dome-like laminectomy group (C2-DOM group, *n* = 16) and C2 partial laminectomy group (C2-PL group, *n* = 16). The cervical curvature (CCI), dura width at C2/3, Japanese orthopedic association (JOA) score, recovery rate (RR), neck disability index (NDI) score, and visual analogue scale (VAS) score were evaluated and compared preoperatively and postoperatively at 1 month, 3 months, 6 months, 1 year, and annually thereafter.

**Results:**

The JOA score and NDI significantly improved at the final follow-up in both groups with no significant intergroup differences. There were no significant differences in preoperative dura width at C2/3 and VAS between the two groups. At the final follow-up, dura width at C2/3 in the C2-PL group was significantly larger than the C2-DOM group, while the VAS of C2-DOM group was significantly lower than C2-PL group. The CCI in both groups decreased compared with before surgery, and there was no significant difference in CCI between the two groups.

**Conclusion:**

C2-DOM is less demolitive and reduces postoperative neck pain, while C2-PL can achieve more adequate decompression without increasing the risk of postoperative cervical kyphosis.

## Introduction

Ossification of the posterior longitudinal ligament (OPLL) of the cervical spine was first reported by a Japanese physician in 1960 (1). It is an ossifying hyperplasia of the posterior longitudinal ligament of the spine, which can be accompanied by severe neurological dysfunction. OPLL is frequently reported in men, in the elderly, and in Asian populations, and its pathogenesis remains elusive. The occurrence and development of OPLL are caused by combination of factors, including genetic factors, endocrine factors, and mechanical stimulation (2, 3) Surgical decompression is required when the ossified posterior longitudinal ligament compresses the cervical spinal cord and causes severe clinical and neurological symptoms. Anterior or posterior surgery both achieved effective decompression of the spinal cord and reduced patients’ neurological symptoms (4). OPLL is mainly located below the C2 segment. For OPLL involving more than three levels and located below the C2 level, C3–7 single open-door laminoplasty or laminectomy with instrumented fusion are the most common posterior surgical options (5). However, the upper cervical OPLL is often identified in cases of continuous type and mixed type of OPLL, and the narrowest space is typically found in the C2–C4 segment (6). Decompression surgery below C2 alone for upper cervical OPLL may lead to inadequate decompression, thus possibly resulting in unsatisfactory surgical outcomes and persistence of neurological symptoms due to C2–C3 stenosis. Therefore, surgical decompression above C2 segment is necessary, although direct decompression through the anterior approach is difficult and risky (6). C2 dome-like laminectomy (C2-DOM) and C2 partial laminectomy (C2-PL) are commonly used posterior approaches of C2 decompression (7–9). However, few studies have compared the efficacy of these two surgical methods. Therefore, the present study aimed to compare the surgical outcomes of C2-DOM with C2-PL for the treatment of upper OPLL and to provide evidence for making clinical decisions.

## Materials and methods

### Patients

This retrospective study included 32 patients who underwent surgery for OPLL of the cervical spine above the C2/3 intervertebral disc at the Third Hospital of Hebei Medical University (Shijiazhuang, China) between January 2016 and January 2020. OPLL was diagnosed based on the computed tomography (CT) and magnetic resonance imaging (MRI) findings for all patients. The inclusion criteria were as follows: (1) Ossified segment of the posterior longitudinal ligament involving the C2 vertebral body and below; (2) C2-DOM or C2-PL (3) Complete preoperative and postoperative follow-up clinical data; (4) Follow-up ≥24 months. The exclusion criteria were as follows: (1) Ossification of the cervical ligamentum flavum; (2) Combination of OPLL of the thoracic and lumbar spine; (3) Patients who were diagnosed with OPLL combined with cervical fractures, deformities, tumors, infections, etc.; (4) History of previous cervical spine surgery.

### Surgical procedures

All surgeries were performed by the same surgical team. After general anesthesia, the patient was placed at a standard prone position and the head was fixed with a skull traction tong. Standard disinfection of the surgical area and sterile draping were performed.

**Figure 1:**
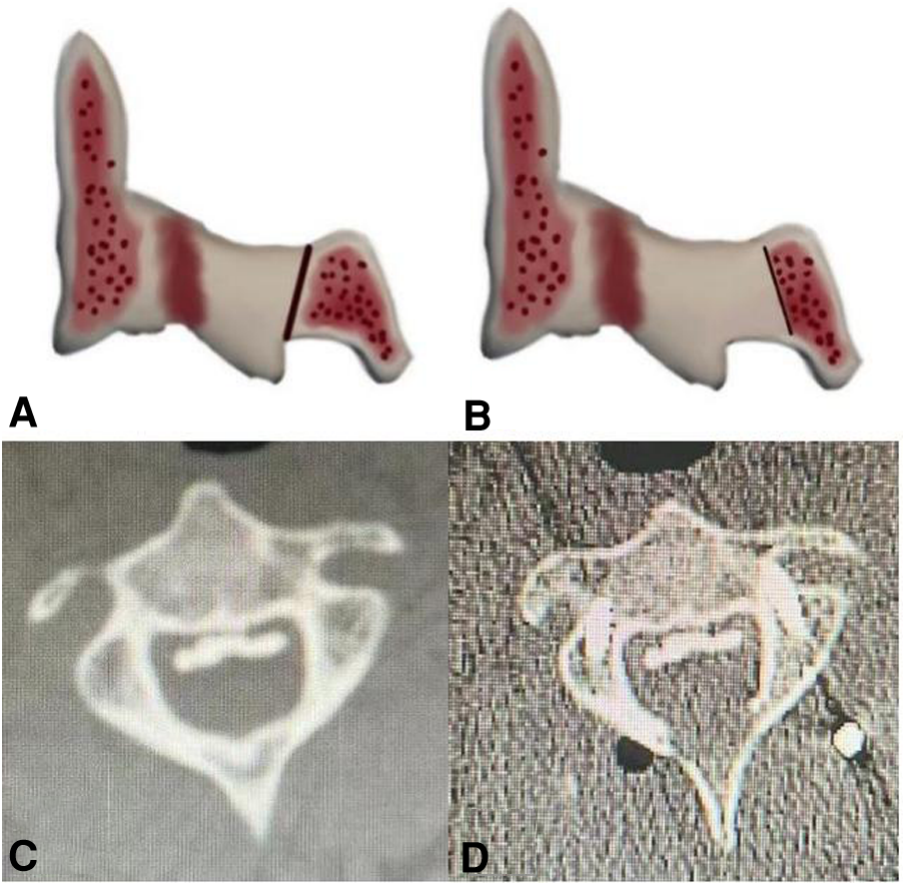
Schematic diagram of C2 dome-like laminectomy (C2-DOM). (**A,B**) The ossification of the posterior longitudinal ligament (OPLL) up to C2. (**C,D**) The C2 after dome-like laminectomy. (the ligamentum flavum and the ventral part of the lamina were removed).

**Figure 2:**
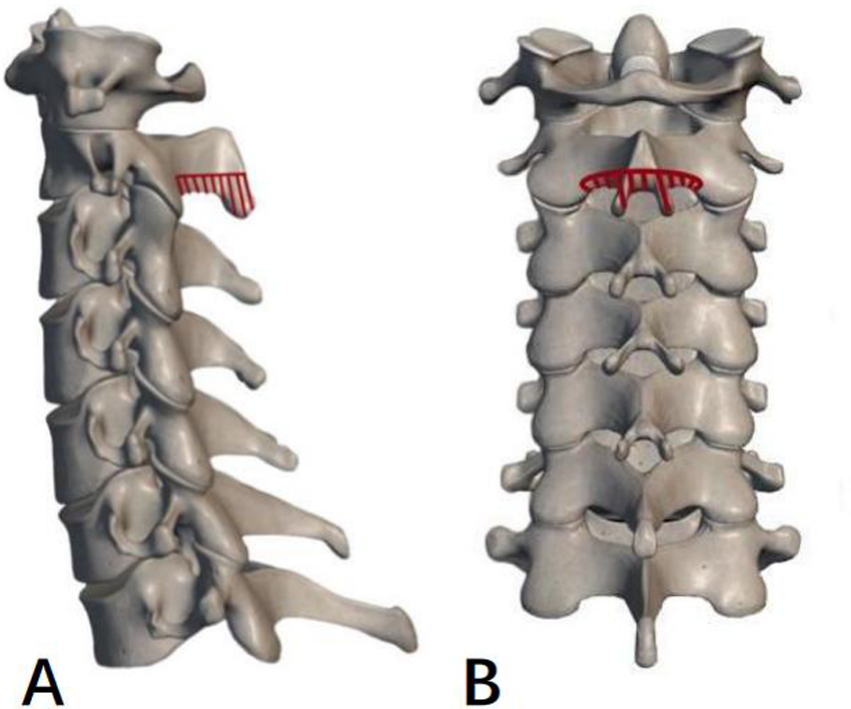
Schematic diagram of C2 partial laminectomy (C2-PL). (**A,B**) The red dotted area is the part of the lamina and spinous process to be resected.

**Figure 3:**
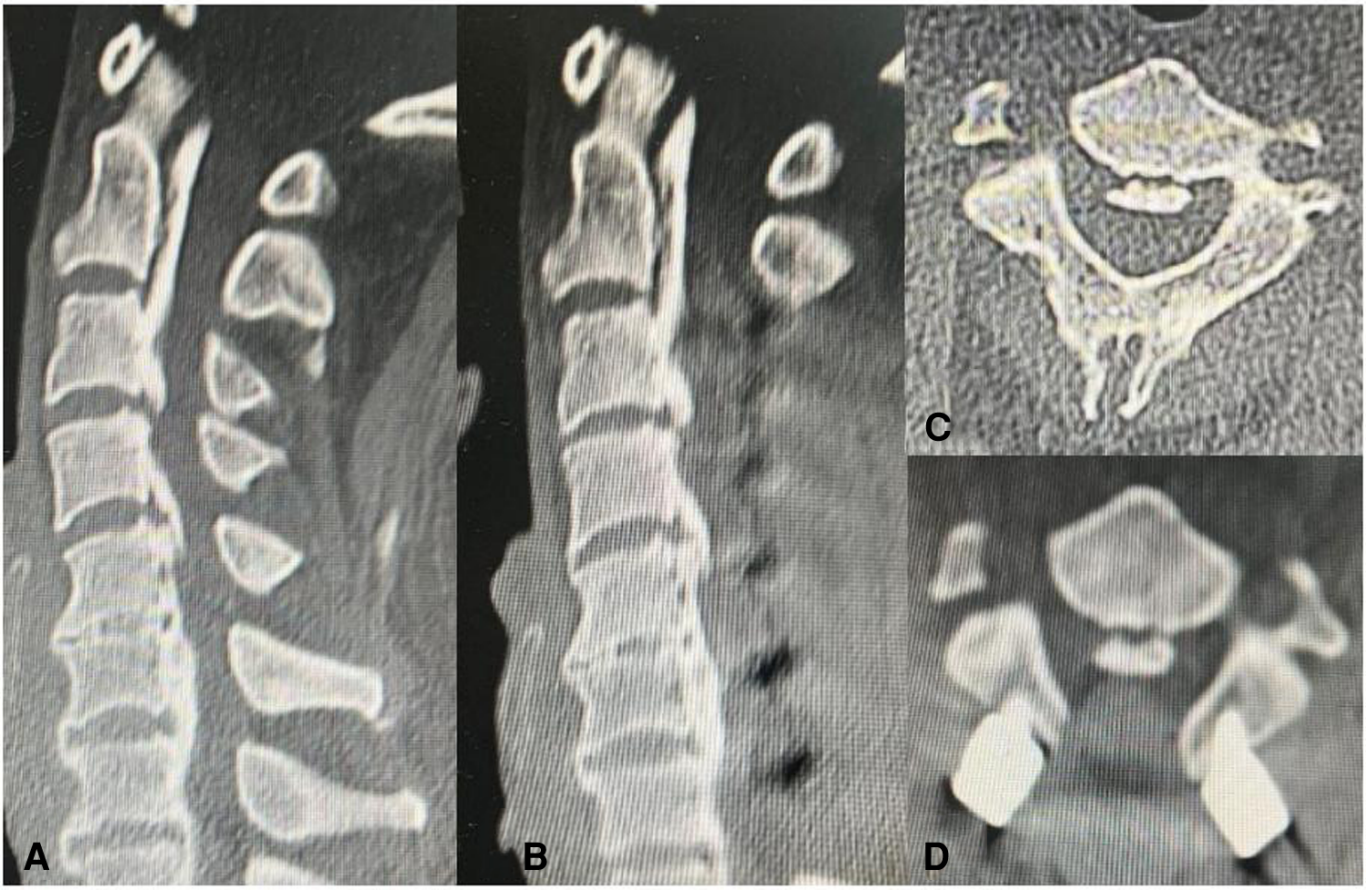
CT scans showing the extent of bone removed by C2 partial laminectomy (C2-PL). (**A,C**) showing the CT image of the cervical spine before the surgery. (**B,D**) showing the CT image of the cervical spine after the surgery.

A midline posterior incision was made between the C2 and T1 spinous processes and paravertebral muscles were dissected to expose posterior elements. All patients underwent standard laminectomy with instrumented fusion from C3 downwards to C7. In the C2-DOM group, a high-speed drill was used to resect a part of the ventral lamina in an arc below the bottom of the C2 spinous process, and the ligamentum flavum was removed until the cervical spinal canal was decompressed. The resection width of the ventral lamina of C2 should be based on the width of the dura mater, and excessive cortical resection should not be performed to avoid C2 spinous process fractures (Figure 1). Eventually cervical paravertebral muscles were reattached to the C2 spinous process. In the C2-PL group, partial laminectomy was performed with a Kerrison rongeur until the lower third or two thirds of the C2 lamina and spinous process were removed (Figure 2). Approximately 5 mm of lamina were removed, along with part of the residual ventral lamina until the dura was decompressed (Figure 3). The ventral ligamentum flavum was removed by Kerrison rongeur until the dura was no longer compressed.

All patients wore a Philadelphia collar for 2–4 weeks after surgery, and then, they started moderately functional exercise of the neck.

### Assessment of outcomes

All patients underwent cervical spine x-ray, CT and MRI preoperatively and postoperatively. The patients were followed up at 1 month, 3 months, 6 months, and 12 months after operation, and once a year thereafter. Clinical outcomes were evaluated during follow-up, and performed x-ray, CT or MRI examinations according to the patient's condition. At last follow-up, the clinical outcomes were evaluated, and x-ray, CT and MRI examinations were performed concurrently. Clinical and radiological outcomes at the last follow-up were used for analysis.

**Figure 4:**
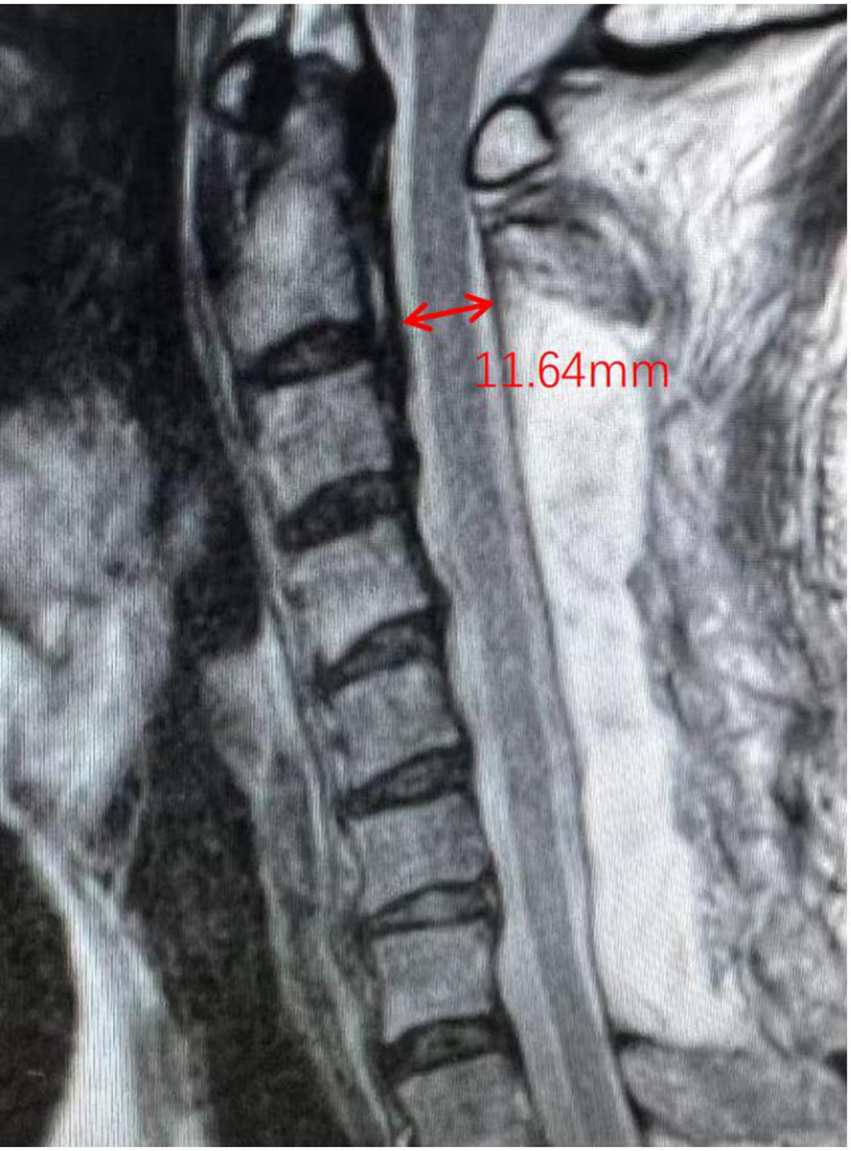
Schematic diagram of C2/3 dura width. The width of C2/3 on MRI scan was evaluated. The dura width in schematic diagram was 11.64 mm.

The distance between the anteroposterior diameter of the dura of C2/3 was assessed on T2-weighted MR cross-sectional images of the cervical spine (Figure 4). The C2–C7 cervical curvature index (CCI) at the last follow-up and preoperative CCI were recorded by cervical lateral x-ray to calculate the changes in lordosis. The ossification type of the posterior longitudinal ligament of the cervical spine was recorded on the lateral CT of the cervical spine. Three independent spinal surgeons, who were not involved in the study, performed radiological measurements, and the average values of all observers were used in the present study.

Neurological function was assessed using the Japanese orthopedic association (JOA) score. The neurological recovery rate (RR) was calculated as follows: recovery rate (%) = (final JOA score—preoperative JOA score)/(17—preoperative JOA score) × 100. The visual analogue scale (VAS) score was used to evaluate axial pain in the posterior cervical region or in the suprascapular region. The functional status of the cervical spine was assessed using the neck disability index (NDI). Three independent spinal surgeons, who were not involved in the study, performed the assessments, and the average values of all observers were used in the present study. Patients' complications, such as cerebrospinal fluid leakage, infection, nerve root palsy, axial neck pain, neurological deterioration, and implant failure were recorded.

### Statistical analysis

Continuous data are presented as means ± standard deviations (SD), while categorical data are shown as absolute frequencies. The Wilk-Shapiro test was used to assess normality of data distribution. The unpaired *t*-test or Mann–Whitney *U* test were used to analyze parametric and nonparametric continuous data, respectively. The Chi-square test was used to analyze categorical data. Paired *t*-test or Wilcoxon test were used for intra-group comparisons of parametric and nonparametric continuous data, respectively. Data were analyzed using SPSS 25.0 software (IBM Corp., Armonk, NY, USA). *P* < 0.05 was considered statistically significant.

## Results

32 patients were involved in this study, including 26 men and 6 women. Patients' age ranged between 45 and 72 years, with a mean age of 57.5 ± 8.4 years old. A total of 29 patients (90.63%) had ossified C2 segment, 3 patients (9.38%) had ossified C1 segment, 10 (31.25%) patients had mixed ossification, 18 (56.25%) patients had continuous ossification, and 4 (12.5%) patients had segmental ossification. All patients were followed up for 2–8 years, with an average of 3.25 years. There were 31 patients with comorbidities, including 13 patients with heart disease, 20 patients with hypertension, 10 patients with cerebrovascular disease, 2 patients with diabetes mellitus, and 2 patients with osteoporosis.

There was no significant difference in demographic data between the two groups of patients (Table 1). As shown in Table 2, there were no significant differences in the type of the OPLL, preoperative C2/3 dura width, and preoperative CCI between the two groups. At the last follow-up, the width of the dura at C2/3 in both groups significantly increased compared with that before surgery (C2-DOM group: 12.6 ± 1.5 mm vs. 7.9 ± 1.9 mm, *P* < 0.001; C2-PL group: 13.5 ± 0.9 mm vs. 7.3 ± 1.5 mm, *P* < 0.001), and the width of the dura at C2/3 in the C2-PL group was significantly larger than that in the C2-DOM group (13.5 ± 0.9 mm vs. 12.6 ± 1.5 mm, *P* < 0.001). At the last follow-up, the CCI value was 17.9 ± 9.4% in C2-DOM group and 15.8 ± 5.2% in C2-PL group with no significant differences (*P* = 0.598).

**Table 1:** Comparison of patient characteristics between C2-DOM group and C2-PL group.

	C2-DOM group (*n* = 16)	C2-PL group (*n* = 16)	*P*-value
Age (years)	57.9 ± 8.7	57.2 ± 8.3	0.812
Gender (male/female)	13/3	13/3	1
Body Mass Index (Kg/m^2^)	30.5 ± 5.2	26.9 ± 4.0	0.112
Follow-up (months)	37.9 ± 14.8	40.3 ± 13.9	0.634
Operation time (minutes)	194.7 ± 83.2	198.4 ± 68.9	0.89
Blood loss (ml)	562.5 ± 387.9	443.8 ± 222.8	0.42

Values are expressed as mean ± standard deviation. C2-DOM group = C2 dome-like laminectomy group. C2-PL group = C2 partial laminectomy group.

**Table 2:** Comparison of radiological measurement between C2-DOM group and C2-PL group.

	C2-DOM group (*n* = 16)	C2-PL group (*n* = 16)	*P*-value
Type of OPLL			0.497
Local	0	0	
Segmental	1	3	
Continuous	9	9	
Mixed	6	4	
**Dura width at C2/3 (mm)**			
Preoperative	7.9 ± 1.9	7.3 ± 1.5	0.366
Last follow-up	12.6 ± 1.5	13.5 ± 0.9	0.043
*P*-value	<0.001	<0.001	
**CCI (%)**			
Preoperative	21.5 ± 7.8	18.5 ± 8.0	0.293
Last follow-up	17.9 ± 9.4	15.8 ± 5.2	0.598
*P*-value	0.071	0.143	

Values are expressed as mean ± standard deviation. C2-DOM group = C2 dome-like laminectomy group. C2-PL group = C2 partial laminectomy group.

OPLL, ossification of posterior longitudinal ligament; CCI, cervical curvature index.

As shown in Table 3, functional outcomes in both groups significantly improved, and there was no significant difference in the preoperative JOA score, NDI score, and VAS score, between the two groups. At the last follow-up, significant improvements in JOA score, NDI score and VAS score were observed in the two groups. There was no significant difference in the JOA score and NDI score between the two groups at the final follow-up. The VAS scores in both groups were increased at the last follow-up (C2-DOM group: 24.6 ± 1.6; C2-PL group: 35.9 ± 1.5), however, the VAS score of C2-DOM group was significantly less than that in C2-PL group (*P* < 0.001).

**Table 3:** Comparison of clinical outcomes between C2-DOM group and C2-PL group.

	C2-DOM group (*n* = 16)	C2-PL group (*n* = 16)	*P*-value
**JOA**			
Preoperative	9.3 ± 2.0	10.2 ± 2.0	0.225
Last follow-up	14.8 ± 1.2	14.7 ± 2.4	0.530
*P*-value	<0.001	<0.001	
RR (%)	72.2	64	0.676
**NDI (%)**			
Preoperative	25.8 ± 10.7	24.8 ± 12.6	0.808
Last follow-up	11.7 ± 4.6	16.0 ± 6.3	0.692
*P*-value	<0.001	0.020	
**VAS**			
Preoperative	22.3 ± 1.5	23.1 ± 2.7	0.348
Last follow-up	24.6 ± 1.6	35.9 ± 1.5	<0.001
*P*-Value	<0.001	<0.001	
**Postoperative Complications [number of patients (percentage)]**			
C5 nerve root palsy	1(6.3%)	0	1
Axial neck pain	1 (6.3%)	3 (18.8%)	0.600
CSF leakage	1 (6.3%)	0	1
Spinal cord injury	0	1 (6.3%)	1
Infection	0	0	
Implant failure	0	0	
Hematoma	0	0	

Values are expressed as mean ± standard deviation. C2-DOM group = C2 dome-like laminectomy group. C2-PL group = C2 partial laminectomy group.

JOA, Japanese orthopedic association; RR, recovery rate; VAS, visual analog scale; NDI, neck disability index; CSF, cerebrospinal fluid leakage.

There was 1 patient in the C2-DOM group who experienced C5 palsy after surgery (*P* = 1), and it was resolved after conservative treatment. In addition, 1 patient in the C2-DOM group and 3 patients in the C2-PL group experienced sustained axial pain after surgery (*P* = 0.600), and they were not significantly improved at the last follow-up. Besides, 1 patient in the C2-DOM group had cerebrospinal fluid leakage (CSF), which resolved at 6 days after surgery when the drainage tube was removed and the incision was sutured under local anesthesia. Moreover, 1 case in the C2-PL group was found with deterioration of bilateral limb muscle strength after recovery from anesthesia. Methylprednisolone was given as a bolus dose of 30 mg/kg in 15 min, followed by a pause of 45 min and a subsequent continuous infusion of 5.4 mg/kg/hour for 23 h. However, muscle strength did not improve, being grade 2/5 according to manual muscle test (MMT) at the last follow-up. No significant differences were found regarding complication rates between the two groups (Table 3). No patients in either group experienced infection, hematoma, implant failure, or other complications after surgery.

## Discussion

The surgical treatment of cervical OPLL includes anterior surgery, posterior surgery, and combination of anterior and posterior surgery. Although anterior surgery can achieve the objective of direct and sufficient decompression (10, 11), the risk of anterior surgery is higher (12, 13).

Kong et al. (14) concluded that the space available at the level cephalad to the stenotic segment is an important predictor of cord postoperative shift. Therefore, when MRI shows compression above the C2/3 intervertebral disc, only the decompression of the segment below C3 may result in the limited posterior translation of the spinal cord and insufficient decompression above the C2/3, which may affect the recovery of neurological function. Therefore, decompression above the C2/3 segment is necessary. Some researchers have also used anterior surgery to decompress the upper cervical spine. Chen et al. (15) reported a case of anterior controllable anti-displacement and fusion (ACAF), and achieved satisfactory recovery after surgery. The surgical technique is complicated, and the extent of surgical decompression cannot be directly observed intraoperatively. Therefore, the posterior approach was selected in the present study.

As for surgical decompression at the C2 level, Takeshita (16) demonstrated that compared with C3–C7 open-door laminoplasty, additional C2 open-door laminoplasty would disrupt the overall balance of the cervical spine and lead to cervical instability. Therefore, no patient underwent C2 laminoplasty in this study. In 1989, Matsuzaki (7) proposed C2 dome-shaped laminoplasty for the treatment of OPLL involving C2, and achieved satisfactory clinical results. Next, in 2016, Japanese scholars reported this lamina-sparing C2 dome-shaped decompression surgical method. While some researchers (9) pointed out that the C2-DOM is complicated, and measuring and reproducing the “dome” size and shape is challenging, C2-PL may also achieve satisfactory clinical results. However, a few studies compared the efficacy of C2-DOM and C2-PL in the treatment of upper cervical OPLL.

In our study, there was no significant difference in the preoperative NDI score, VAS score, and the recovery of neurological function between the two groups. The VAS scores in the C2-DOM group were significantly better than those in the C2-PL group after surgery (Table 3). Research suggested that disruption of the C2 spinous process, the attachment of the semispinalis cervicalis, and the semispinalis capitis muscle, as well as surgery involving the C7 segment, may cause or aggravate postoperative neck pain (17, 18). Shunsuke et al. found that the decrease in the strength of the deep extensor muscles of the neck after surgery was resulted in an imbalance of the extensor and flexor muscles at the cervical spine, which was highly correlated with axial symptoms (19). C2-PL removes a part of the lamina and C2 spinous process, disrupting the attachment of muscles and ligaments, while C2-DOM only partially removes the ventral structure of the C2 lamina and preserves the C2 spinous process. Through C2-DOM, not only the C2 segment is fully decompressed and the backward shift distance of the spinal cord increases, but also decreases the surgical damage to posterior neck muscles reducing the incidence of postoperative neck pain. According to the results of the present study, C2-DOM is superior to C2-PL in terms of postoperative axial symptoms.

In the present study, the CCI was measured to evaluate cervical lordosis in the two groups. Excessive destruction of posterior facet joint and muscle ligament structure, especially muscle attachment at C2 segment, was reported to be associated with postoperative cervical kyphosis and deterioration of neurological function (20). Biomechanical and clinical studies have shown that preservation of the semispinalis muscle could reduce the incidence of cervical kyphosis and stabilize the cervical spine (9). Liu et al. (21) reported that as the majority of patients had continuous OPLL located behind the C2 and C3 vertebral bodies, the lordotic effect might reduce the incidence of segmental kyphosis after surgery. Therefore, they suspected that C2 single-door laminoplasty could not increase the incidence of postoperative cervical kyphosis. Yu et al. (22) showed that there was no significant difference in the results of CCI at the last follow-up between the two groups of patients who underwent C2 single-door surgery or C2-DOM. In the present study, postoperative CCI in the C2-PL group was not significantly different from that in the C2-DOM group with less damage to the C2 muscle attachment, which could be related to the fact that the majority of patients in the C2-PL groups had continuous OPLL located behind the C2 and C3 vertebral bodies, and bony structure maintained the cervical lordosis, which could also be attributed to the small sample size of this study.

The postoperative dura width at C2/3 in the C2-DOM group was 12.6 mm, and the postoperative C2/3 dura width in the C2-PL group was 13.5 mm. C2-PL achieved decompression of the dorsal side of the C2 spinal cord by partially removing the lamina and C2 spinous process. The C2-DOM could retain the original shape of the C2 vertebral body *via* partially removing the bone and adhering ligament tissue on the ventral side of the C2 lamina. Therefore, the decompression of the C2 segment in the C2-PL is more thorough than the dome-like decompression of the C2 laminectomy. When the ossification above the C2/3 occupies a large area of the spinal canal, C2-PL can achieve a more adequate decompression. Overall, C2-DOM led to less postoperative axial symptoms in patients, and C2-PL was more efficacious in expanding the effective spinal cord space. C2-PL can achieve more adequate decompression when the ossification above the C2/3 level occupies a large space in the spinal canal.

This study has some limitations. First, the number of patients included in this study was small due to the low incidence of upper OPLL. Second, the follow-up period was short, with an average of 39 months, thus, the long-term clinical efficacy needs to be further evaluated. Furthermore, the small sample size and short follow-up time might lead to inaccurate radiological measurements, especially for the incidence of cervical kyphosis. Finally, this was a retrospective study, and there might be retrospective bias in data collection. Therefore, further multicenter, prospective, randomized controlled study should be conducted for further validation.

## Conclusions

Both C2-DOM and C2-PL can treat patients with upper OPLL and achieve effective decompression. C2-DOM has less damage and lower postoperative neck pain, while C2-PL possesses more advantages in terms of expanding the spinal canal, and the risk of cervical kyphosis is comparable to that of C2-DOM.

## Data Availability

The original contributions presented in the study are included in the article/Supplementary Material, further inquiries can be directed to the corresponding author/s.
